# Rethinking Maintenance Terminology for an Industry 4.0 Future

**DOI:** 10.36001/ijphm.2021.v12i1.2932

**Published:** 2021

**Authors:** Melinda Hodkiewicz, Sarah Lukens, Michael P. Brundage, Thurston Sexton

**Affiliations:** 1 Faculty of Engineering and Mathematical Sciences, University of Western Australia, Perth, 6009, Australia; 2 GE Digital, San Ramon, CA, 94583, USA; 3 National Institute of Standards and Technology, Gaithersburg, MD 20899, USA; 4 National Institute of Standards and Technology, Gaithersburg, MD 20899, USA

## Abstract

Sensors and mathematical models have been used since the 1990’s to assess the health of systems and diagnose anomalous behavior. The advent of the Internet of Things (IoT) increases the range of assets on which data can be collected cost effectively. Cloud-computing and the wider availability of data and models are democratizing the implementation of prognostic health (PHM) technologies. Together, these advancements and other Industry 4.0 developments are creating a paradigm shift in how maintenance work is planned and executed. In this new future, maintenance will be initiated once a potential failure has been detected (using PHM) and thus completed before a functional failure has occurred. Thus corrective work is required since corrective work is defined as “work done to restore the function of an asset after failure or when failure is imminent.” Many metrics for measuring the effectiveness of maintenance work management are grounded in a negative perspective of corrective work and do not clearly capture work arising from condition monitoring and predictive modeling investments. In this paper, we use case studies to demonstrate the need to rethink maintenance terminology. The outcomes of this work include 1) definitions to be used for consistent evaluation of work management performance in an Industry 4.0 future and 2) recommendations to improve detection of work related to PHM activities.

## 1. Introduction

The promise of Prognostics and Health Management’s (PHM’s) impact on maintenance management performance has yet to be fulfilled. Large amounts of money have been spent, starting with the US Government’s investments in the Joint Strike Fighter program (Birkler, Graser, Arena, Cook, & Lee, 2001) and continuing with significant European Union funding (Hartly, Brauer, & Dunne, 2004) as well as unknown amounts of corporate funds, yet published examples of integrated and effective maintenance management programs seamlessly incorporating appropriate PHM approaches are few. Why is this?

PHM work processes are concerned with making intelligent, informed and appropriate decisions about maintenance and logistics actions based on diagnostics and/or prognostics information, available resources, and operational demand (Vachtsevanos, Lewis, Roemer, Hess, & Wu, 2006). A PHM program must fit into an organization’s maintenance management system. Successful management systems depend on the ability to integrate strategy and planning with support, execution, performance evaluation and improvement processes. This requires having the necessary leadership and stakeholder commitments in place. We argue that PHM is overly focused on the technical aspects of health monitoring and evaluation, such as which sensors and models to use, and insufficiently focused on how the results of these analyses integrate into the work management system necessary to execute the proposed remedial measures.

The major elements of the PHM work process include how data is collected (manually taking measurements and the use of sensors, including Internet of Things (IOT) sensing system), predictive modeling capabilities (models and/or rules which use the collected data), and the processes of initiating action based on the information extracted from the measured data, and then validating if the prediction was correct. The output of this process includes recommendations to execute maintenance work based on an assessment of risk and cost of failure and determining what tasks to perform, such as replacing, repairing, or overhauling the component proactively before the failure event occurs. Data-driven analytics are often used to determine the timing for the work, such as estimating when a component will fail. The suitability of a PHM approach depends on the failure behavior of the component and the consequence of failure. Only certain components meet this qualification. Ensuring the right strategy is being applied to each failure mode of each component is a key factor in the success both of the PHM process and maintenance management in general (Lukens & Markham, 2018). Reliability-centered maintenance (RCM) is a widely-used, systematic and risk-based approach to preserve system function by identifying an appropriate maintenance strategy for an asset and its components. RCM decision logic guides the user towards one of the following strategies: a) use-based also known as fixed-interval restoration/repair (FIR) strategy, b) strategy-based on condition monitoring and inspections, c) failure-finding and d) run-to-failure maintenance strategies (SAE, 2009), (IEC, 2016).

The business case for applying PHM comes from demonstrating that the benefits outweigh the costs to implement and sustain. Benefits from PHM may include lowered maintenance costs, from less over-maintaining, and reduction of production losses incurred from unscheduled downtime. Such benefits are realized only when the full process is executed. In other words, the business value of predictive models is dependent on the effectiveness of the maintenance organization in responding and taking action to the outputs of the models. PHM programs are costly to run and complex to manage. They require data, either from sensors and/or manual collection, but also require highly trained analysts and reliability engineers. Failure behavior is stochastic, so the analysis always involves a number of uncertainties, with false-positive recommendations leading to additional costs and loss of trust (Böhm, 2013; Crocker, 1999). Companies investing in implementing PHM technologies in their organizations need to measure the effectiveness of the investment. Since PHM is part of the maintenance management program, consideration needs to be given to compare PHM-inspired work with existing FIR and run-to-failure strategies, while considering all the resources needed to manage the different programs. We suggest that one of the key reasons PHM has taken so long to implement, and struggles to be part of “business-as-usual” in the maintenance workflow, is the people and processes involved are not well integrated into the maintenance management system and costs, benefits, and performance measures for the PHM program are not clearly identifiable and tracked. These claims and solutions to address them are explored in this paper.

The paper is organized as follows. First, we describe the data and experience on which the paper is based. This discussion is followed by a description of the maintenance terms and the maintenance work management process and some the challenges arising from these definitions as industry moves to online data collection with IoT sensors and deployment of predictive analytics models. These challenges are then illustrated with case studies drawn from industry data. Finally, we propose some solutions to the issues through 1) a repackaging of corrective maintenance work, 2) activation of a detection method code, and 3) generation of a predictive analytics modeling work type. We close with commentary on how these proposed changes will positively impact work management metrics and support organizations that are embracing PHM to evaluate the success of their PHM programs. To assist readers we have included a list of the nomenclature at the end of the paper.

## 2. DATA

The data on which this paper is based is held confidentially in the Computerized Maintenance Management Systems (CMMS) and Enterprise Asset Management (EAM) Systems of more than a hundred organizations across many sectors. The authors acknowledge the privileged access they have to these data due to their professional roles as academics, government and corporate researchers and data scientists. No company data or names are used in this paper but examples inspired by transactions in the data sets are provided for illustrative purposes. To protect proprietary information, all variables in the examples and case studies have been anonymized or simulated based on the actual observations made from the data.

## 3. The current situation

### 3.1. Maintenance terms and definitions

Over the years a central tenet of maintenance has been that a well-managed maintenance organization will be ‘in control’ of their maintenance process. This means there is a limited number of unexpected failures and most of the work of the maintainers is executed in a structured and organized way that is cost-effective and makes efficient use of available resources. To determine how maintenance departments are performing, managers need a way of coding raw maintenance data and calculating metrics. There are many standards and best practices for maintenance terms (EFNMS, 2007; ISO, 2016; Kelly, 2006; NERC, 2020; SMRP, 2017) and very little agreement within and between sectors as to how terms are used or how the data collected contribute to work management metrics. We use two guides in this paper: 1) Society of Maintenance and Reliability Professionals (SMRP) Best Practices Maintenance and Reliability Body of Knowledge and 2) ISO 14224 Petroleum, Petrochemical And Natural Gas Industries - Collection And Exchange Of Reliability And Maintenance Data For Equipment. The SMRP Best Practices Maintenance and Reliability Body of Knowledge, now in its 5th Edition (SMRP, 2017), has a set of work management metrics. ISO 14224 provides guidelines for collecting maintenance data in a standard format, including recommendations for structuring a functional location hierarchy, taxonomic definitions with respect to equipment boundaries, codes for corrective work events, failure modes, mechanisms and causes and suggested maintenance metrics (ISO, 2016).

### 3.2. Work management

**Figure 1.**
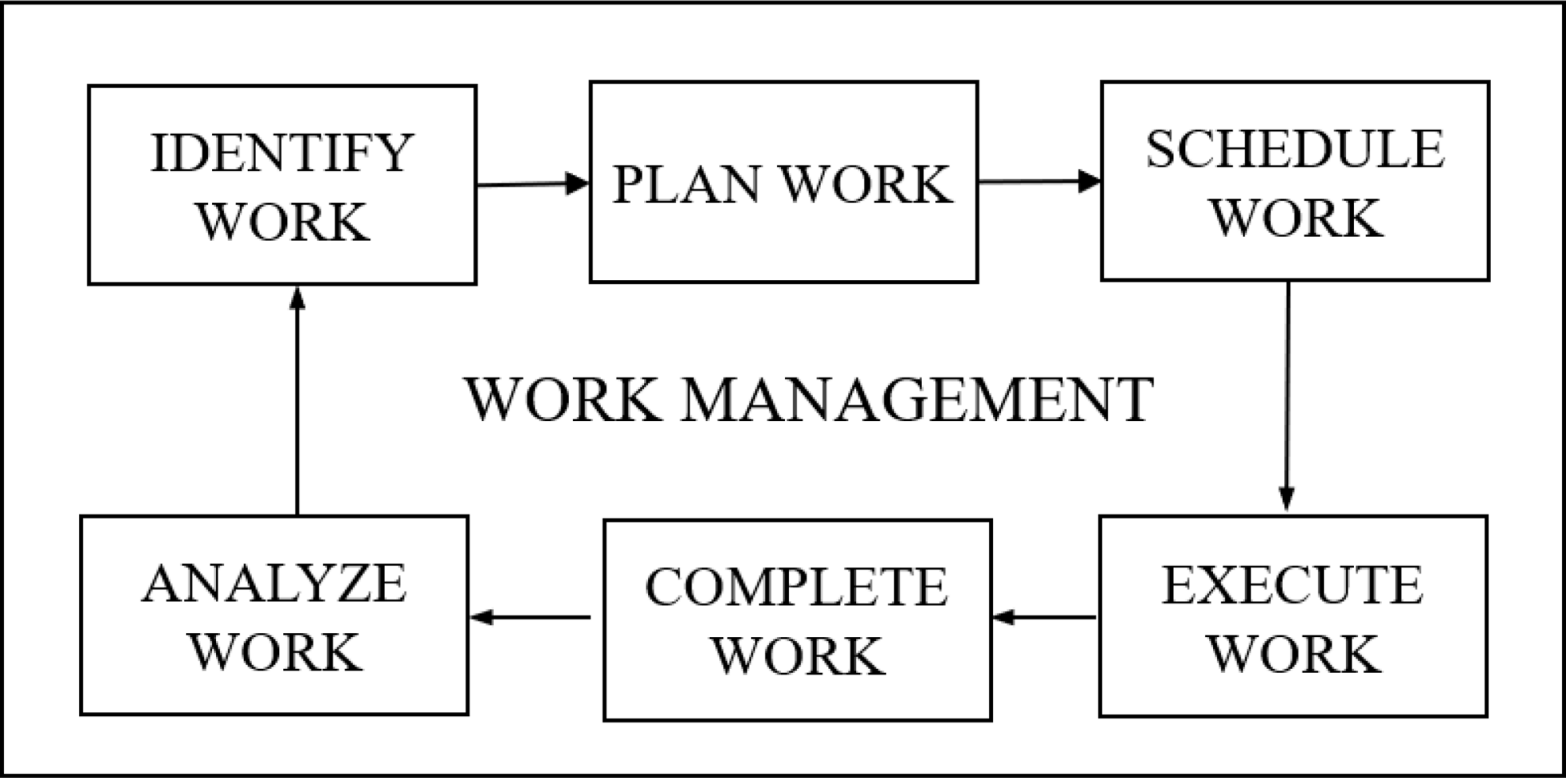
The six stages of maintenance work management

An effective maintenance organization needs to have a well-managed work management process. Work done on every asset is associated with a work order record, and this record moves through the six stages shown in Figure 1 where work is 1) identified, 2) planned, 3) scheduled, 4) executed, 5) completed and 6) analyzed. In large organizations, as many of 10,000 work orders per month are managed by computerized maintenance management systems (CMMS).

The CMMS provides a centralized location for the data on each stage of the work management process to be created, managed, and stored by the different stakeholders. Stakeholders with roles in the creation and execution of work order data are coordinators, planners, schedulers, craft supervisors and the maintenance technicians (Gulati & Smith, 2009).

The work management process, shown in Figure 1, is broadly the same across every maintenance organization. However, the way in which the data is collected, labelled and stored varies widely from company to company. CMMS databases are set up and used differently by each company and even from site to site within the same organization. These differences range from the selection of which data fields get populated, to the structuring of the equipment taxonomies, hierarchies and codes which get used, to the design of the database schema underlying the process. Furthermore, it is common for there to be wide variation of the quality of the entered data in terms of accuracy and completeness (Hodkiewicz & Ho, 2016), (Lukens, Naik, Saetia, & Hu, 2019), (Naik & Saetia, 2018).

### 3.3. Structured and corrective work

The SMRP Best Practice Guideline defines the following work types: *Structured work*, *Corrective work*, *Improvement & Modification work* and *Other work*. Our focus in this paper is on *Structured work* and *Corrective work* as they account for approximately 80% of all maintenance work (SMRP, 2017).

*Structured work* is maintenance labor used on planned and programmed routines for preventative maintenance (PM) tasks (restoration and replacement at fixed intervals regardless of condition) and predictive maintenance (PdM) tasks (labor used to assess the condition of an asset to determine the likelihood of failure before actual failure occurs) (SMRP, 2017). Both PM and PdM tasks are identified from Original Equipment Manufacturer’s (OEM) recommendations and strategy development decision trees such as Reliability-Centered Maintenance that take into account failure behavior and consequence as discussed earlier. For completeness, we also mention that Failure Finding (or Functional Testing) is also a form of structured work. This neither predicts nor prevents equipment failures but tests otherwise hidden functions, such as protective devices and systems, to see whether they have already failed. The rules for deciding how and when to do these tests/inspections are quite different to the rules used to decide how often to apply PM or PdM tasks and not the focus of this paper.

*Corrective work* is done to restore the function of an asset after failure or when failure is imminent (SMRP, 2017). Ideally corrective work is actioned in a controlled manner prior to failure as a result of the predictive tasks generated by structured work such as inspections and asset condition data, and associated with the use of models and data analytics. Rather than spending time on structured work, many industries spend an undesirably large proportion of maintenance labor hours on unexpected corrective work, in a fix-when-it-breaks culture where work is actioned after the failure has occurred either because a maintenance strategy is absent or inappropriate or is in place but has not been actioned effectively. The definitions used in this paper for these, and other, terms are shown in Table 1.

### 3.4. Perceptions that all corrective maintenance is bad

Many maintenance textbooks were written in the 1990s and 2000’s (Coetzee, 1997; Márquez, 2007), long before PHM became as salient as it is today. In these books the term corrective work is principally associated with a run-to-failure maintenance strategy rather than work resulting from a PdM strategy, which uses condition data and analytics to detect the presence of a potential failure. More recent maintenance textbooks do note that corrective work can be the result of PdM activities (Gulati & Smith, 2009), but do not elaborate on the potential for predictive analytical models to generate corrective maintenance work. As PHM has matured, a need now exists to differentiate between ‘good’ corrective and ‘bad’ corrective work. Specifically, we consider the different impacts types of corrective work have on a maintenance organization and in this framework propose new performance measures of work management.

### 3.5. Confusion in use of terms to describe PM, CBM and PdM work

SMRP defines condition-based maintenance (CBM) as an “equipment maintenance strategy based on measuring the condition of equipment against known standards in order to assess whether the equipment will fail during some future period and then taking appropriate action to avoid the consequences of that failure.” We note that “taking appropriate action” is corrective maintenance and needs to be tracked separately to CBM related work. SMRP agrees with this notion as it defines “corrective work from structured work” as maintenance labor used on corrective work that was identified through preventive and/or predictive maintenance tasks and completed prior to failure in order to restore the function of an asset” (SMRP, 2017). However, data on corrective work from structured work is rarely collected and reflected in work management metrics in practice.

In SMRP preventative maintenance work (PM) is defined as “actions performed on a time- or machine-run-based schedule that detect, preclude or mitigate degradation of a component or system”. In other words, preventative work includes FIR work and the work involved in collecting vibration, thickness, temperature and other condition data. In our experience, this definition creates confusion between what is PM and what is predictive maintenance (PdM) work.

In the remainder of this paper, we take preventative maintenance (PM) to encompass work done under an FIR strategy. We adhere to the ISO 14224 definition of predictive maintenance (PdM), which includes maintenance involved in inspections, tests and periodic condition monitoring. This is consistent with the SMRP Best Practice guide as it provides identical definitions to CBM and PdM work and explicitly says that the terms CBM, on-condition maintenance, and PdM can be used interchangeably.

**Figure 2.**
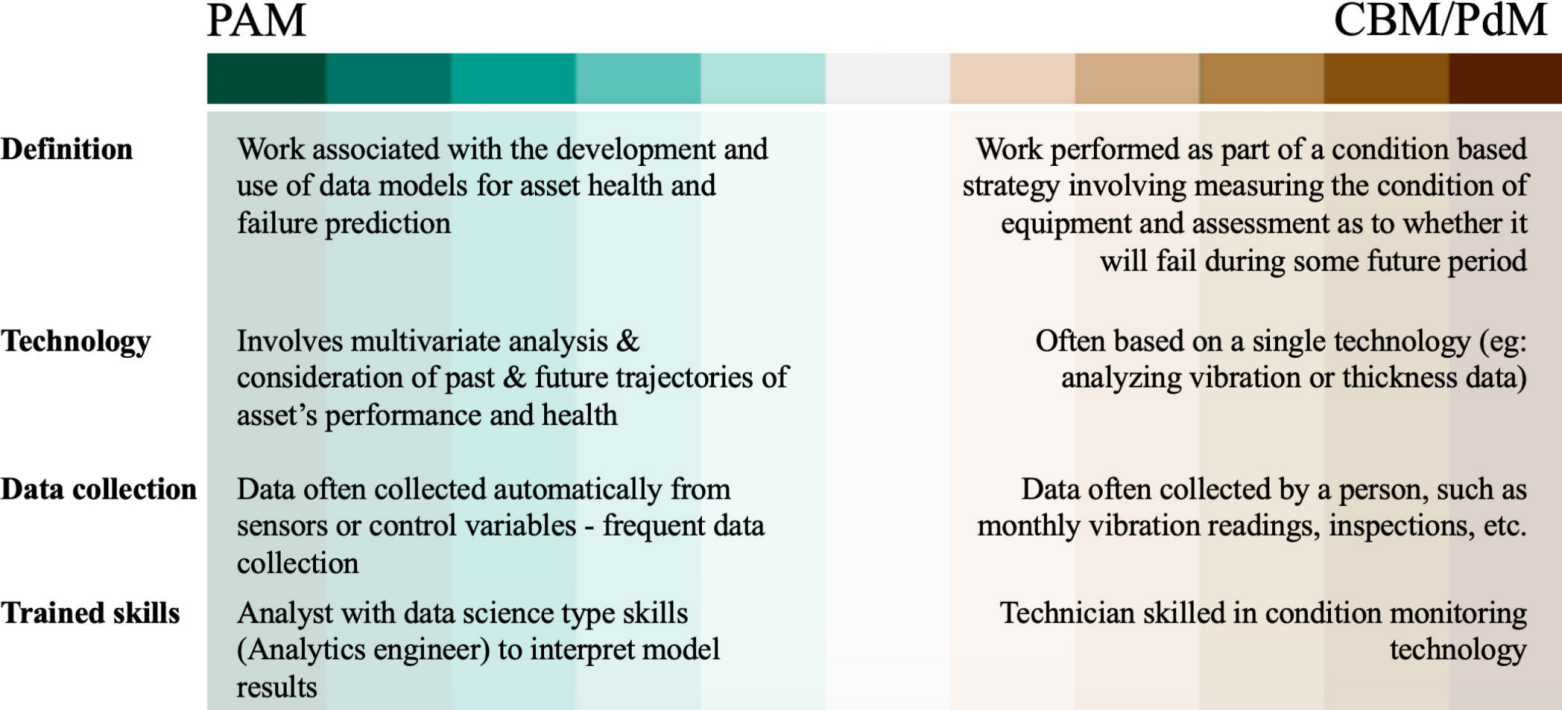
Distinguishing between activities involved in PAM and CBM/PdM. The classification is not discrete as some activities may fall on the continuum between the different work types

Finally, we propose a new work type called Predictive Analytics Modeling (PAM) work. This work is associated with the development and use of data models for asset health and failure prediction. The increasing use of sensing systems, analytics and the use of cloud systems is changing the way analytics is being performed (Kwon, Hodkiewicz, Fan, Shibutani, & Pecht, 2016). The nature of the analysis, often combining data from multiple systems and sensors, is very different from the way CBM work is traditionally performed. CBM work is often based on a single technology, e.g., analyzing vibration or thickness data and comparing to a predefined threshold. PAM work involves multivariate analysis and consideration of the past and future trajectory of the asset’s performance and health profile. In defining PAM we recognize that some activities fall on a continuum between PAM and CBM/PdM, which is illustrated in Figure 2.

Examples of PAM work types are shown in the blue box in Figure 4. We propose the PAM code be used to capture both the work involved in running the modeling programs as well as the recommendations arising from them. Corrective work resulting from PAM programs can then be measured and evaluated.

### 3.6. Maintenance metrics and invisibility of maintenance work initiated from predictive analytics (PAM)

A list of maintenance work management metrics related to PM and PdM work is provided in Table 2. The metrics in this table are drawn from SMRP Best Practices and ISO 14224, (SMRP, 2017), (ISO, 2016). We draw your attention to the following. First, no category exists for work done as a result of the maintenance predictive analytics team (PAM work). Currently, we suspect PAM work is rolled into PdM work in both the SMRP and ISO 14224 metrics. Given the increased investment in PAM, we suggest it merits its own definition, as shown in Table 1 and its own metric in Table 2 so that the outcomes from investments in PAM can be measured and tracked. Second, observe that in SMRP, the PM and PdM activities are lumped together, whereas in ISO 14224 they are separate.

## 4. Example case studies

### 4.1. Case study 1: Evaluating traditional work management metrics on PAM work

This case compares the evaluation of traditional performance measures for different maintenance strategies for bearings on the same make/model/duty pump. Bearing failure on a pump, if not acted on, can lead to seizure in the shaft and damage to the housing as well as unplanned downtime incurring operational losses. Pump A follows an FIR plan on time-based intervals. Every 6 months, the CMMS generates a work order to replace the bearings. Pump B has a PdM strategy using a condition-based approach based on monthly, manually-collected vibration readings. Pump C utilizes a PAM approach based on a model using vibration and temperature sensors installed on the pump, historical reliability analysis, as well as pressure and flow data from the control system. In a two year period, Pump A had four 6-month bearing replacements, while Pumps B and C had 2 on-condition replacements. We illustrate the costs and metrics resulting from these three strategies over 2 years in Table 3.

The following assumptions are made: the parts costs and labor rates are identical, the required parts are kept in stock and that there is a standard work procedure (SWP) for bearing replacement. In the cases of Pumps B and C we assume there is an inspection made after a fault is identified to confirm the fault and scope the work required. This inspection work is classed as corrective as is the work related to changing out the bearing as it occurs after a failure has initiated.

In practice, using this data to calculate metrics (such as metrics from SMRP) requires additional information such as knowing if the inspection work to confirm the potential fault was planned or unplanned, how far in advance the fault was detected on Pumps B and C, and if the resulting work to replace the bearing was planned or unplanned, scheduled or unscheduled. We assume that the bearing replacement work for Pumps B and C is unscheduled, meaning insufficient notice was given after the fault was detected to get the work onto the weekly schedule. Over 2 years, Pump A has 32 scheduled hours and zero unscheduled hours. Pump B has 6 scheduled hours (from the condition assessment activities), and 18 hours of unscheduled work from the replacement activities. Pump C has zero scheduled hours (because the condition assessments are automatically collected) and 18 unscheduled hours.

In this case, commonly-used work management metrics around scheduling, such as schedule compliance (measure of adherence to the maintenance schedule) and reactive work (proportion of work hours that are unscheduled), would reward the Pump A strategy over that for Pump B and C. Like-wise, Pumps B and C would have high values for corrective work (proportion of work hours that are corrective) and low values for metrics measuring structured work.

A few representative metrics comparing the values between the 3 pumps evaluated for the bearing replacement work are shown in Table 4. Observe that when the maintenance costs and total hours are considered a different picture emerges. Over 2 years, Pump A is the most costly strategy at $4800, compared to Pump B at $3200 and Pump C at $2600.

There are some additional points to observe from this case study. In this example, maintenance costs are calculated from labor and parts costs, which is common industry practice. The costs for PdM and PAM work on Pumps B and C do not include the costs of the condition monitoring equipment or the costs of analysts and reliability engineers involved. Also, it was assumed that there were no events when a fault should have been detected but was not.

Another point to observe is the trade-off that exists when evaluating the effectiveness of a PHM strategy against other possible strategies. Often comparisons are one-dimensional, such as marketing claims about the benefits of PHM as compared to a run-to-failure strategy (Schleichert, Bringmann, Kremer, Zablotskiy, & Köpfer, 2017), and the benefits of PHM against an FIR strategy through reducing the potential to waste useful life of components by too-frequent replacement. In reality, there are several different strategy options and each has strengths and weaknesses which need to be systematically reviewed. We can see from the case above that some current work management metrics strongly favor FIR strategies. To see the benefits of PHM in the work management metrics we need to rethink how corrective maintenance is classified and weigh the complexities introduced by PHM on the work management process, as shown in Figure 5.

### 4.2. Case study 2 - The need to classify corrective work for measuring the effectiveness of a PHM initiative

The second study steps through the requirements needed for data from the CMMS if the end goal is to understand and evaluate the effectiveness of a PHM initiative. The first requirement is the ability to differentiate work related to a PHM initiative from other work. Once this step is completed, measures for tracking and measuring work effectiveness such as using metrics from Table 2 can be deployed. ISO 14224 recommends capturing how a failure was detected when creating a work order through a field called ”Detection Method”. In general, the CMMS needs to be configured for capturing detection method information, however, in practice, this information is seldom captured or is unreliably populated.

The variation of different work order type codes across a handful of industrial companies is illustrated in Table 5, ranging from as few as 3 to as many as 20 work order types. No matter how many of these codes there are, the bulk of work orders contain links to codes that are clearly some form of “Corrective Work” as well as structured work that is either “Preventative Work (PM)” or “Predictive Work (PdM)”.

The fourth column in Table 5 shows where detection method information could be inferred - which could range from not being used in the CMMS implementation at all to a large number of possible codes to pick from (which may be the case if Detection Method is integrated with Request Type codes). Finally the last column shows how in many cases, the detection field is unused or missing in the CMMS system. The purpose of this exercise was to show that while there is large variation in how work order type and detection information is typically captured from company to company, ultimately, in practice, the same 2–3 work order codes are used most of the time and the detection code is seldom used if at all.

If a detection method field is populated, the impact of a PHM initiave can be tracked. We look more in-depth at an industrial company who applied a PHM program through application of a PAM strategy to 10 critical pumps at one plant. As part of this initiative, the company also implemented practices to mark if a corrective work event was the result of a predictive model alert or not when creating a notification.

**Figure 3.**
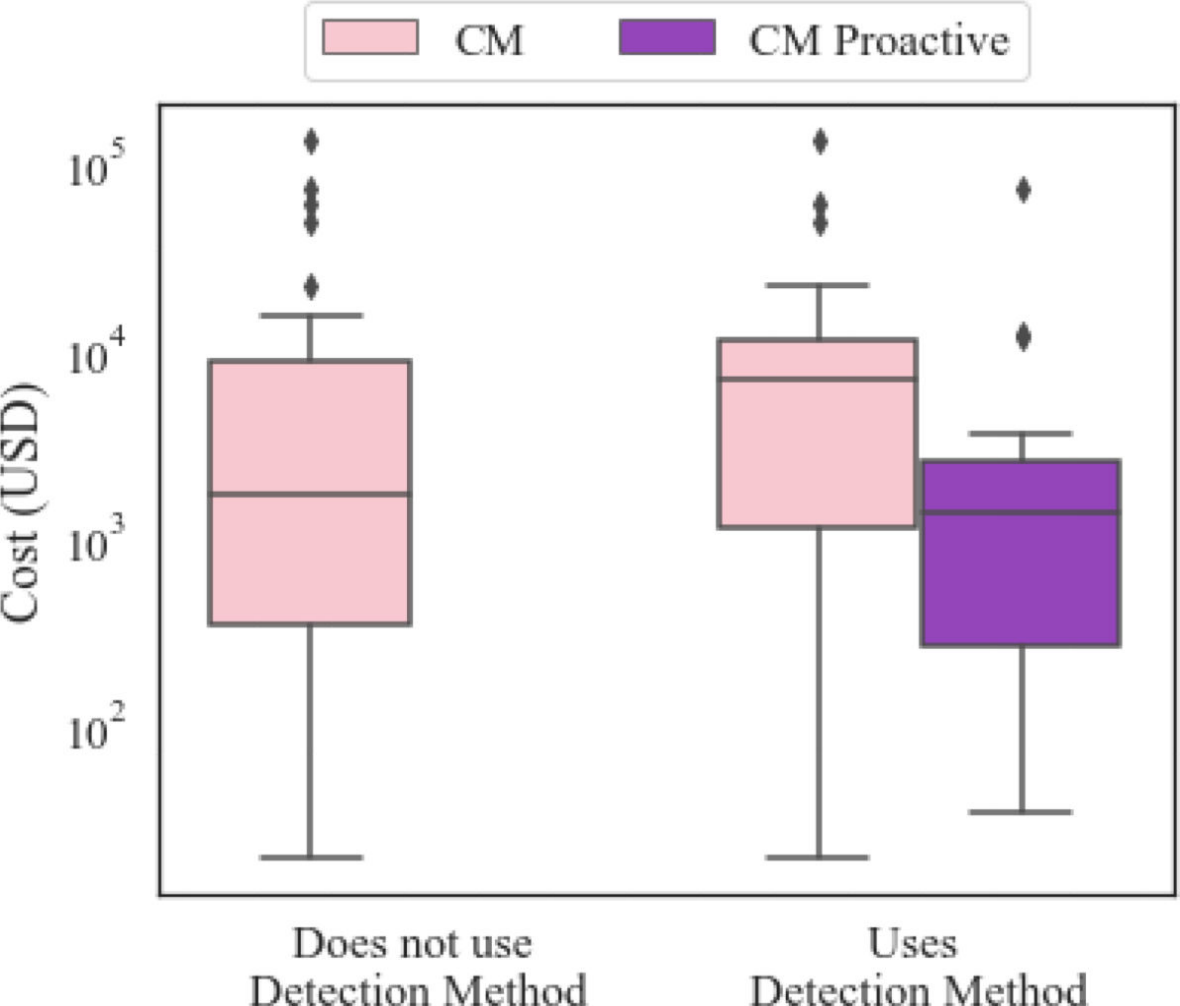
Comparison of corrective maintenance (CM) costs when the detection method field is not used (left) and when it is used (right). CM costs are depicted in pink and costs from CM work that was detected proactively are in purple. If the company did not record detection method information for CM work (as in common practice), they would only know the CM work costs (left case).

After 2 years, maintenance data on these 10 pumps was queried from the CMMS. There were 45 total corrective work events over the 2-year window for the 10 pumps, evenly distributed between corrective work events that were unanticipated and corrective work events which were generated from an alert from the predictive models. Boxplot representations of the maintenance costs, which are comprised of costs from parts and labor, are shown in Figure 3. The case on the left summarizes all of the corrective costs, while the case on the right shows a summary of the costs split between corrective work events and corrective work events which were detected proactively.

Even though the event frequencies are similar, the contribution of costs from the corrective work events were significantly greater. This difference is due to the issues associated with not having labor, parts and plans readily available for the unanticipated work as well as the scope of work often being greater due to the damage caused. These numbers do not include the effects of any downtime.

There are two main takeaways from this case study. First, unanticipated corrective work and corrective work that is detected proactively may have different population characteristics and should be differentiated when using the data such as for measuring the effectiveness of a PHM initiative or for resource planning. The other key takeaway is that in order to even make this differentiation, a detection field needs to be utilized in the CMMS.

## 5. Repackaging Corrective maintenance

**Figure 4.**
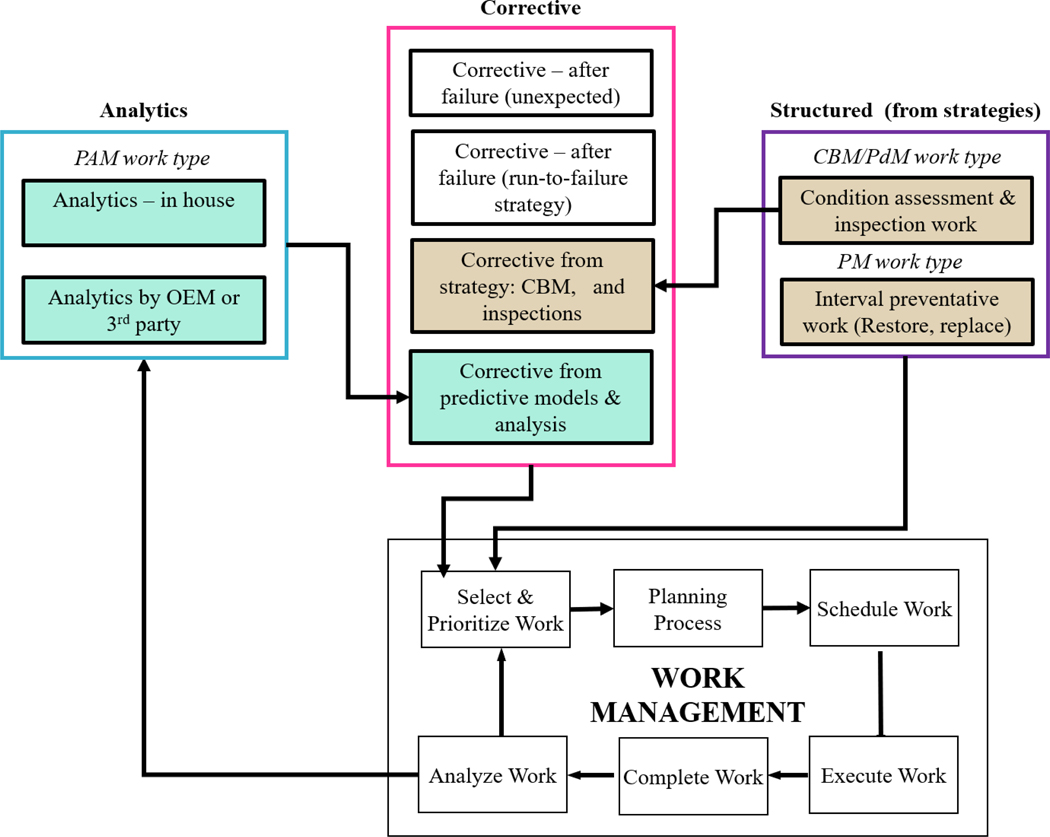
Maintenance Management 4.0 showing integration between corrective, structured and analytics work identification, PdM (Predictive Maintenance), PM (Preventative Maintenance), PAM (Predictive Analytics Modeling) work types and the Work Management System

Recall that corrective work is defined by SMRP as “work done to restore the function of an asset after failure or when failure is imminent.” We suggest “Corrective work” needs to stop being a catch-all expression and be repackaged as specific work types. What defines the corrective work types is governed by the maintenance strategy for predefined failure modes. Our solution for repackaging corrective maintenance is shown in Figure 4 and the proposed types of corrective work are summarized in Table 6.

This proposal involves recognizing that the four types of corrective maintenance: a) corrective work after an unexpected failure, b) corrective work after a failure which is expected as the asset has a run-to-failure strategy for that failure mode (e.g., a light bulb), c) corrective work resulting from failures identified during scheduled activities initiated from inspection and condition-based maintenance strategies (e.g., from oil analysis, thermography and periodic vibration monitoring programs), and d) corrective work from predictive models and analysis drawing on data from continuously monitored systems (e.g., statistical and machine learning models). This last category is expected to grow significantly as investments are made in IoT and data analytics capabilities. These four proposed corrective maintenance types are shown inside a pink boundary in Figure 4.

**Figure 5.**
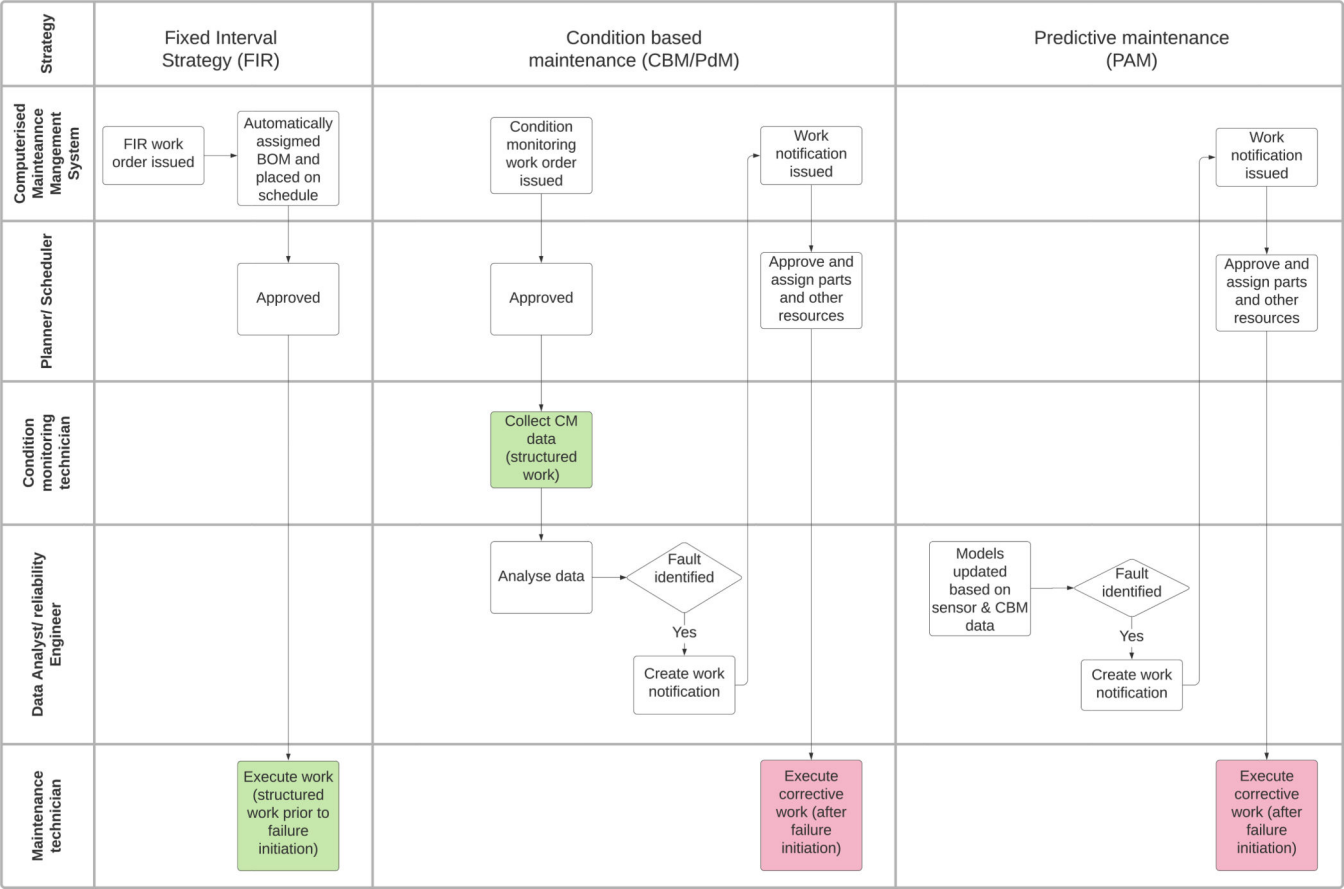
Roles involved in execution of three maintenance strategies: fixed interval (FIR), condition based and predictive. Red boxes signify corrective work orders and green boxes signify structured work orders. A detection code differentiates corrective work that is generated from PdM.

The proposed groupings allow maintenance managers to track the success (or otherwise) of their maintenance strategy. Currently, as shown in Figure 5, corrective work resulting from scheduled condition-based maintenance and predictive maintenance is counted as corrective. Whereas this type of corrective work is in-line with the maintenance strategy and, as shown in Table 3, may result in reduced interventions and lower maintenance costs. Right now the effects of this work are almost impossible to observe through metrics collected in the CMMS unless special steps such as the use of detection method codes are taken, as shown in case study 2.

Once “Corrective work” is no longer used as a catch-all, it is obvious that a set of detection method codes are necessary to distinguish between the different types of corrective work. Specifically, as we start to invest in analysts to develop predictive models, we need to distinguish between this modeling work and work arising from inspections, testing, and periodic condition monitoring, confusingly called Predictive Maintenance (PdM) in the literature.

## 6. Using Detection Method codes

A detection code is the “method or activity by which a failure is discovered” and a selection of examples are given in Table 7 (ISO, 2016). We propose an addition to this list to account for work done by the data analytics team to predict failure using predictive modeling.

While we celebrate the availability of standardized detection codes, as shown in Table 7, we have found the following issues: a) sometimes the CMMS is not configured to collect detection method on the work notification; b) when there is a field on the work notification, it is not filled; c) when the field is filled with a code, the codes are not used consistently; and d) there is no auditing or performance measures for data quality. As a result, there is limited visibility or trust in their use, as was illustrated in the second case study. The use of a detection method code is necessary in order to split the corrective work bucket into a) corrective work resulting from unexpected failure and run to failure strategy, b) corrective work resulting from PdM inspections and condition data collection, c) corrective work resulting from PAM. Note that we propose that there is only a single detection code for corrective work resulting from unexpected failure and run to failure strategy. This is because most corrective failure work requests that arise in these categories will be initiated by either plant operators or possibly maintenance technicians, and they are unlikely to know if there is already a strategy in place to address this failure mode.

The failure to distinguish between different types of corrective maintenance work can lead to the challenges illustrated by the case study 1 in Table 4. Pump A, with an FIR maintenance strategy has zero corrective maintenance hours but the highest maintenance cost. Pumps B and C with PdM and PAM strategies respectively have significant corrective and reactive hours but lower costs.

One of the reasons detection codes for corrective maintenance are important is shown in Figure 5. The first column shows roles involved in execution of structured work under an FIR strategy. The CMMS generates the work order, it passes through the planner and onto the schedule (not shown) from whence it is executed by the maintenance technician. This work is then tagged as structured work as per the SMRP definition (we have colored this green). However, for PdM work only the initial data collection stage is structured work, any work that results from the analysis is by definition corrective work, as the failure has already been initiated. The same goes for PAM work. Both of these are also by definition corrective work. Without detection codes, work arising from condition based and predictive work is often tagged as corrective work in maintenance management systems.

To make matters worse, tracking work arising from condition monitoring, non-destructive testing and other inspections is further complicated since frequently specialist internal or external groups are involved and their work is not always captured on PdM work orders. Manual spot checks, often a feature of vibration, thermography and oil analysis programs may also not be recorded in the CMMS. It can therefore be hard to get an understanding both of the effort being expended on the predictive program and its results. Similar challenges affect the PAM program.

However, since all work resulting from PdM and PAM programs have to be actioned by a maintenance technician and hence need a work order, the solution is to use detection codes. We suggest that every corrective maintenance work order should have a detection code. Of course, one could use the codes suggested in the ISO 14224 guidelines. However, experience has taught us that giving planners, maintenance technicians and operators multiple codes to use (there are 11 listed in ISO 14224) does not work in practice and leads to data quality issues (Brundage, M.P., Sexton, T., Hodkiewicz, M., Morris, K.C., Arinez, J., Ameri, F., Ni, J. & Xiao, G., 2019; Molina, Unsworth, Hodkiewicz, & Adriasola, 2013; Unsworth, Adriasola, Johnston-Billings, Dmitrieva, & Hodkiewicz, 2011; Unsworth, Yeo, & Beck, 2014).

We suggest a simpler system for detection method based on three detection classes as follows: 1) Detection - unexpected, 2) Detection - from structured work activities, and 3) Detection - by analytical prediction. These are an abstraction of the detection methods recommended in ISO along with the proposed new PAM detection method. They are shown in Table 8.

## 7. Rethinking the impact of complexity in executing PdM and PAM work

Maintenance managers, like all managers, operate in an environment of resource constraints. They have to execute work with a fixed pool of maintenance technicians, parts, and tools, equipment and ancillary equipment has to be available when required, and trained technicians can only perform certain work on certain equipment. In this context, structured work is preferred because it is routine and predictable, while corrective work is not.

Planning resources required to move a work order to the execution phase are significantly reduced for structured work because structured work typically has SWPs or “model work orders” available in the CMMS/EAM database containing work instructions along with parts and resources estimates. Corrective work lacks SWPs and as a result each corrective work order needs someone to decide what work is required, what parts and tools are needed, and when it should or could be executed.

When corrective work is actioned depends on factors such as the impact of an asset on production and the severity of the failure event. If an asset that is critical to production fails, often the planning and scheduling phases (shown in Figure 1) are skipped for immediate execution of the work by the maintenance supervisor and crew. If the work is not urgent, then corrective work can pass through the work management process. In either case if corrective work has to be actioned immediately, there is an increased workload for the planner and/or maintenance team. In the latter case, resources originally committed to structured work then need to be redeployed to the corrective work.

Another less appreciated factor in the introduction of PdM and PAM programs is the additional complexities introduced into the system, as shown in Figure 5.

We can see that the end-to-end process for executing PM work has less steps and engages fewer roles than either PdM or PAM work. With an FIR strategy, the work order is generated automatically in the CMMS, and in mature organisations, the Bill of Materials (BOM) and other resources are already assigned. As a result, work orders generated from FIR strategy take little time for the planner to approve and push the work order onto the schedule for future execution. Because the work is executed prior to a failure being initiated, the work is not classed as corrective (shown as green in Figure 5).

However, when we look at PdM and PAM programs, a different picture emerges. Excluding the scheduler, there are only two steps and two roles involved in the execution of maintenance from an FIR strategy compared to eight steps and four roles for PdM and PAM strategies. For PdM programs, vibration, oil, temperature and other data are collected; and when limits are breached, work notifications are submitted to take action. To run PdM programs, the reliability engineer must have assessed the failure modes of the assets and determined that the potential failure can be detected and be detected in time to take action. PAM programs involve data from a number of potential sources being used in statistical models to estimate the remaining useful life. To run PAM programs, both data analysts and condition monitoring technicians require advanced training and the support of specialist tools and/or software, which are often hidden costs for having these strategies.

## 8. Rethinking work management metrics for organizations executing PdM and PAM work

The following is a preliminary list of ideas to guide development of an expanded set of work management metrics for a future where PdM and PAM work will be more prevalent and embedded in maintenance organizations. Our aim is not to argue over current measures of work management performance that looks at control of the work management process, effectiveness of the scheduling process, accuracy of the planning function, overall maintenance cost, resource utilization and other commonly used metrics, but to make the case for additional metrics based on our proposed corrective maintenance definitions and the use of detection method codes. Work to develop and test a suite of work management metrics leveraging the ideas proposed here will be done in a future paper.

Low levels of corrective work are widely seen as desirable and yet values for corrective work are increasing since they currently include work arising from PdM and PM programs. We propose that metrics for corrective work should exclude corrective work resulting from PM inspections, PdM and PAM programs. The use of detection method codes will be necessary to tag the data appropriately with corrective work associated with detection codes resulting from “scheduled activity” and “predictive analytics”.

One way of ensuring maintenance work effectiveness is to eliminate ineffective inspections, tests, and condition-based data collection. To achieve this goal, we should be looking for PM/PdM work orders that have no follow up corrective from PM/PdM work orders. To action this, the work order number of the PM/PdM work will need to be captured on the work order for the “corrective work - scheduled activity” resulting from it.

Establishing the effectiveness of PdM and PAM programs requires a two-step process. First, a corrective work order needs to be executed as a result of the work (as described in the prior paragraph) and second validation that the PdM/PAM analysis was correct and not a false-positive needs to occur. This second step is more complex to manage and will be the subject of future work. Associated with this process are metrics associated with false-negative calls in PdM/PAM when the analysts fail to predict the corrective event.

It is important to know if work is performed in a well coordinated, planned manner (i.e., was it Planned and Scheduled, Planned but not Scheduled, Scheduled but not Planned, or neither Planned or Scheduled). A code for this can only be captured when the job is closed out. Most CMMS already have a Planned/Unplanned/Breakdown type code that can be adapted for this purpose. As mentioned earlier, the ideal situation is to have the majority of work being properly planned and scheduled, regardless of how the work was initiated. If we have PAM-initiated work that is still not able to be conducted in a planned and scheduled manner, then this could be used to trigger a review of whether the PAM model for that failure mode is adequate or appropriate. This data would support a new set of metrics to support more work being Planned and Scheduled regardless of how it is initiated.

Finally, we note that a commonly used metric is maintenance budget compliance. This metric compares planned versus actual maintenance spend at a given frequency (monthly, quarterly and yearly) (SMRP, 2017). Many maintenance managers may be reluctant to move work from structured to PdM and PAM work because this leads to uncertainty in when work will occur. Structured work occurs at set times and in line with budget expectations hence is predictable from a budget compliance perspective. Corrective work resulting from PdM and PAM programs will always have uncertainty associated with when it occurs. Costs associated with work initiated by PdM and PAM are therefore less predictable, both in quantum and timing. These variances between budget and actual spend in a specific time period result in difficult conversations with accountants and general managers who do not necessarily understand this new model for maintenance. A more sophisticated approach to assessing budget compliance that recognizes this situation is required if PdM and PAM programs are to be successful.

## 9. Summary of recommendations and conclusions

As organizations invest in implementing PHM technologies, they need a way to measure the effectiveness of the investment. Current standard and best practices maintenance metrics and definitions are insufficient moving forward in an Industry 4.0 world. It is extremely challenging to extract the required information to determine if corrective work is the result of an unexpected failure, part of a run-to-failure strategy, from CBM and inspections or as a result of predictive analysis from maintenance work order data as it is captured today. In this paper, we have demonstrated how current definitions of corrective maintenance result in an under-appreciation of PdM and PAM work. We propose a new set of three classes of corrective work. Alongside this proposition, we propose the use of a simple detection method code to assist with the determination of the class of corrective work. We also draw attention to the under-appreciation of the complexities and high costs of additional roles necessary to run PdM and PAM programs. Our aim in this paper is to stimulate discussion in the community about the issues raised here in the hope that a new paradigm, designed to accommodate a Maintenance 4.0 world with a greater proportion of work from PdM and PAM activities can emerge.

## Figures and Tables

**Table 1.** Definitions for different types of maintenance work used in this paper

Input	Definition
Corrective work	Maintenance work done to restore the function of an asset after failure or when failure is imminent
Structured work	Maintenance labor used on planned, programmed routines such as Preventive Maintenance tasks and Predictive Maintenance routes
Preventative maintenance (PM) work	Work performed as part of a fixed interval replace, repair or restore maintenance strategy. PM work encompasses work done under a fixed-interval restoration/repair (FIR) strategy.
Predictive maintenance (PdM) work	Work performed as part of a condition-based strategy involving measuring the condition of equipment and assessment as to whether it will fail during some future period
Predictive Analytics modeling (PAM)	Work associated with the development and use of data models for asset health and failure prediction

**Table 2.** Work Management Key Performance Indicators (KPIs) relating to preventative (PM) and predictive (PdM) maintenance work from SMRP guide and ISO 14224.

Metric	Definition
**SMRP**	
PM & PdM Work Order Compliance	Count of PM & PdM work orders completed within the report date range
PM & PdM Yield	Volume of corrective work that results directly from PM and PdM work orders
PM & PdM Effectiveness	The amount of corrective work identified from PM/PdM work orders that was truly necessary
PM & PdM Compliance	PM and PdM work order execution and completion compliance against criteria such as by required date
**ISO 14224**	
PM overdue	Volume of PM work orders overdue
PdM overdue	Volume of PdM work orders overdue
PdM complete	Number of PdM data collection activities complete

**Table 3.** Case study 1: Comparison of the impact on work management metrics by fixed interval (FIR), condition-based and predictive work for bearing replacements over a 2 year period

Inputs	Pump (Strategy)		

	A (FIR)	B (PdM)	C (PAM)
Number of replacement events	4	2	2
**Labor Hours**			
Structured work to replace bearings	32	–	–
Monthly CBM data collection (structured)	–	12	–
Corrective inspection	0	2	2
Corrective work to replace bearings	–	16	16
Structured maintenance	32	6	–
Corrective maintenance	–	18	18
**Cost** ($USD)			
Parts costs	$1 600	$800	$800
Structured maintenance	$3 200	$600	–
Corrective maintenance	–	$1 800	$1 600
Total	$4 800	$3 200	$2 600

**Table 4.** Case study 1: Comparison of Work management metrics evaluated for the three strategies using the values reported in Table 3. Values in bold indicate the “best” value across each of the three cases.

Metric	FISR	PdM	PAM
**SMRP Metric (%)**			
Corrective Maintenance Hours	**0**	67	89
Corrective Maintenance Cost	**0**	56	62
Reactive Work Hours	**0**	75	100
**Totals**			
Maintenance Cost	$4 800	$3 200	**$2 600**
Hours	32	24	**18**

**Table 5.** Example of the number of work order types, detection codes and number of work orders in which detection method codes are missing

Company	Work Order Type Codes (#)	Work Order clearly Corrective, PM, or PdM (%)	Detection Method codes (#)	Detection Method Missing (%)
A	8	96	–	–
B	20	92	20	70
C	10	90	10	60
D	10	96	10	98
E	3	99	10	96

**Table 6.** Mapping between different types of corrective maintenance work, detection method from ISO 14224, and those who initiate the work notification

Type of corrective maintenance	ISO 14224 detection method	Work notification initiator
Corrective - *work occurring after a failure — unexpected and unwanted*	No detection code	Maintainer or operator
Corrective - *work that is anticipated, as the asset has a run to failure strategy*	No detection code	Maintainer or operator
Corrective - *work from condition based maintenance and inspections as per CBM strategy*	Work arising from scheduled activities	Maintainer, operator or condition monitoring technician
Corrective - *work arising from analysis and predictive models based on data (PAM)*	Work arising from continuous monitoring	Reliability engineer or data analyst

**Table 7.** Detection methods from ISO 14224 (ISO, 2016)

Detection name	Description
Periodic maintenance	Failure discovered during preventative service, replacement or overhaul when executing the preventative maintenance program.
Inspection	Failure discovered during planned inspection, e.g. visual inspection, non-destructive testing.
Functional testing	Failure discovered by activating an intended function and comparing against a predefined standard.
Periodic condition monitoring	Failures revealed during planned condition monitoring of a predefined failure mode, either manually or automatically. e.g. thermography, vibration, oil analysis, sampling.
Continuous condition monitoring	Failures revealed during a continuous condition monitoring of a pre-defined failure mode.
Corrective maintenance	Failure observed during corrective maintenance.
Casual observation	Casual observation during routine or casual operator checks, mainly by senses (noise, smell, smoke, leakage, etc.)
On-demand	Failure discovered during an on-demand attempt to activate an equipment unit.
**Predictive Analytics Modeling (PAM)**	Failure predicted as a result of predictive analytics work.

**Table 8.** Proposed detection methods

Detection class	Includes detection methods
Unexpected	Detection during corrective maintenance, by casual observation and on demand
Structured activity	Detection during periodic maintenance, inspection, functional testing, periodic condition monitoring
Predictive analytics	Detection predicted by analytics (includes data from continuous condition monitoring)

## Nomenclature

BOM: Bill of Materials
CBM: Condition-based Maintenance
CM: Corrective Maintenance
CMMS: Computerized Maintenance Management Systems
EAM: Enterprise Asset Management
FIR: Fixed-interval repair, restore, replace
OEM: Original Equipment Manufacturer
PAM: Predictive Analytics Modeling
PdM: Predictive Maintenance
PM: Preventative Maintenance
RCM: Reliability-centered Maintenance
SMRP: Society of Maintenance and Reliability Professionals
SWP: Standard Work Procedure

## References

[R1] Birkler J, Graser JC, Arena MV, Cook CR, & Lee G. (2001). Assessing competitive strategies for the joint strike fighter: Opportunities and options (Tech. Rep.). California, USA: Rand National Defense Research Inst.

[R2] Böhm T. (2013). How precise has fault detection to be? Answers from an economical point of view. In 26th International Congress on Condition Monitoring and Diagnostics Engineering Management (comadem 2013). Helsinki, Finland.

[R3] Brundage MP, Sexton T, Hodkiewicz M, Morris KC, Arinez J, Ameri F, Ni J. & Xiao G. (2019). Where do we start? Guidance for technology implementation in maintenance management for manufacturing. Journal of Manufacturing Science and Engineering, 141(9). 10.1115/1.4044105 PMC11494710 39439469

[R4] Coetzee J. (1997). Maintenance. Pretoria, South Africa: Trafford Publishing.

[R5] Crocker J. (1999). Effectiveness of maintenance. Journal of Quality in Maintenance Engineering, 5, 307–313.

[R6] EFNMS. (2007). Maintenance Key Performance Indicators (Standard No. En 15341). The European Federation of National Maintenance Societies.

[R7] Gulati R, & Smith R. (2009). Maintenance and reliability best practices (2nd ed.). Industrial Press Inc.

[R8] Hartly K, Brauer P, & Dunne J. (2004). Offsets and the Joint Strike Fighter in the UK and the Netherlands. In Arms trade and economic development: Theory, policy and cases in arms trade offsets (pp. 118–125). London: Routledge.

[R9] Hodkiewicz M, & Ho MT-W (2016). Cleaning historical maintenance work order data for reliability analysis. Journal of Quality in Maintenance Engineering, 22(2), 146–163.

[R10] IEC. (2016). Dependability management – Maintenance and maintenance support (Standard No. AS IEC 60300.3.14). Geneva, Switzerland: International Electrotechnical Commission.

[R11] ISO. (2016). Petroleum, petrochemical and natural gas industries – Collection and exchange of reliability and maintenance data for equipment (Standard No. ISO14224:2016). Geneva, Switzerland: International Organization for Standardization.

[R12] Kelly A. (2006). Maintenance systems and documentation. Elsevier.

[R13] Kwon D, Hodkiewicz MR, Fan J, Shibutani T, & Pecht MG (2016). IoT-based prognostics and systems health management for industrial applications. IEEE Access, 4, 3659–3670.

[R14] Lukens S, & Markham M. (2018). Data-driven application of PHM to asset strategies. In Proceedings of the annual conference of the prognostics and health management society (Vol. 10).

[R15] Lukens S, Naik M, Saetia K, & Hu X. (2019). Best practices framework for improving maintenance data quality to enable asset performance analytics. In Proceedings of the Annual Conference of the PHM Society. Scottsdale, AZ.

[R16] Márquez AC (2007). The maintenance management framework: models and methods for complex systems maintenance. London, UK: Springer.

[R17] Molina R, Unsworth K, Hodkiewicz M, & Adriasola E. (2013). Are managerial pressure, technological control and intrinsic motivation effective in improving data quality? Reliability Engineering & System Safety, 119, 26–34.

[R18] Naik M, & Saetia K. (2018). Improving data quality by using best practices and cognitive analytics. In SMRP conference proceedings. Orlando, FL.

[R19] NERC. (2020). Generating Availability Data System - Data Reporting Instructions (Standard). Atlanta, GA.

[R20] SAE. (2009). Evaluation Criteria for Reliability-Centered Maintenance (RCM) Processes (Standard No. JA1011–2009). SAE International.

[R21] Schleichert OP, Bringmann B, Kremer H, Zablotskiy S, & Köpfer D. (2017, July). Predictive maintenance: Taking pro-active measures based on advanced data analytics to predict and avoid machine failure [White Paper]. Retrieved from https://www2.deloitte.com/content/dam/Deloitte/de/Documents/deloitte-analytics/Deloitte\%5FPredictive-Maintenance\%5FPositionPaper.pdf

[R22] SMRP. (2017). Society of Maintenance and Reliability Professionals (SMRP) Best Practices 5th. Edition (Vol. 5; Standard). Atlanta, GA: Society for Maintenance and Reliability Professionals.

[R23] Unsworth K, Adriasola E, Johnston-Billings A, Dmitrieva A, & Hodkiewicz M. (2011). Goal hierarchy: Improving asset data quality by improving motivation. Reliability Engineering & System Safety, 96(11), 1474–1481.

[R24] Unsworth K, Yeo G, & Beck J. (2014). Multiple goals. Journal of Organizational Behavior, 35(8), 1064–1078.

[R25] Vachtsevanos G, Lewis F, Roemer M, Hess A, & Wu B. (2006). Intelligent fault diagnosis and prognosis for engineering systems. Hoboken, New Jersey: Wiley.

